# Sleep Disorders in Early Psychosis: Incidence, Severity, and Association With Clinical Symptoms

**DOI:** 10.1093/schbul/sby129

**Published:** 2018-09-08

**Authors:** Sarah Reeve, Bryony Sheaves, Daniel Freeman

**Affiliations:** 1 Department of Psychiatry, University of Oxford, Warneford Hospital, Oxford, UK; 2 Oxford Health NHS Foundation Trust, Oxford, UK

**Keywords:** insomnia, nightmares, schizophrenia, comorbidity

## Abstract

Sleep disturbance is known to be associated with psychosis, but sleep disorders (eg, insomnia, nightmare disorder, sleep apnea) have rarely been investigated. We aimed to provide the first detailed assessment of sleep disorders and their correlates in patients with early psychosis. Sixty outpatients aged between 18 and 30 with nonaffective psychosis were assessed for sleep disorder presence, severity, and treatment using a structured diagnostic interview, sleep diaries, and actigraphy. Psychotic experiences, mood, and psychological wellbeing were also measured. Forty-eight patients (80%) had at least one sleep disorder, with insomnia and nightmare disorder being the most common. Comorbidity of sleep disorders within this group was high, with an average of 3.3 sleep disorders per patient. Over half of the sleep disorders had been discussed with a clinician but almost three-quarters had received no treatment. Treatment according to clinical guidelines was rare, occurring in only 8% of cases (*n* = 13). Sleep disorders were significantly associated with increased psychotic experiences, depression, anxiety, fatigue, and lower quality of life. Sleep disorders are very common in patients with psychosis, may have wide-ranging negative effects, and merit routine assessment and treatment in psychiatric practice.

## Introduction

Over recent years, there has been a growing recognition of the potential importance of disrupted sleep in psychosis.^1,2^ Sleep disturbance is a primary motivation for many patients to seek mental health treatment,^3,4^ and there is an awareness among both professionals and patients of an interaction between poor sleep and worsening mental health.^5,6^ Many studies have found that sleep disturbance (eg, shorter sleep time, lowered sleep quality) is associated with increased psychotic experiences in clinical,^7^ nonclinical,^8^ and at-risk^9,10^ populations.^1,2^ Yet relatively few studies have investigated clinical sleep disorders (eg, insomnia, nightmare disorder, sleep apnea) in psychosis, which clearly cause sleep disturbance and therefore present targets for intervention.^1,11^ This study sought to carry out the first detailed assessment of clinical sleep disorders in patients experiencing early psychosis.

The evidence to date does indicate that sleep disorders are an important clinical issue in psychosis. Insomnia, by far the most researched sleep disorder, has been shown to be common, severe, and—importantly—treatable in patients with psychosis.^12,13^ There are also indications that other sleep disorders, for instance, nightmares and circadian disruption, may be more common in psychosis.^11,14,15^ Only one study identified had assessed multiple sleep disorders, finding that comorbid insomnia and nightmares in schizophrenia was associated with a greatly elevated suicide risk above either sleep disorder individually.^16^ To date, therefore, the range of potential sleep disorder comorbidity in psychosis has not been fully elucidated, despite its possible clinical importance.

Taking sleep disorders in psychosis seriously may have important benefits. Recent manipulation studies have demonstrated that simulating insomnia increases psychotic experiences,^17^ and, conversely, treating insomnia reduces psychotic experiences.^18^ Therefore, independently of the importance of treating sleep disorders in their own right,^19^ treating sleep represents a new therapeutic target for psychosis itself. Yet a recent survey of clinicians found that the use of formal sleep assessments with patients with psychosis is rare, as is provision of evidence-based sleep treatments.^5^

We set out to report on the presence, severity, and treatment of a wide range of sleep disorders in patients with nonaffective psychosis attending outpatient clinical services. The focus was on patients with early psychosis, to limit the impact of age and long-term medication usage. The association between sleep disorders, psychiatric symptoms, and wellbeing was tested as a secondary objective. We hypothesized that: sleep disorders are common, severe, and often unassessed and untreated among patients with psychosis; and that patients with sleep disorders in the context of psychosis have more severe psychotic experiences, lowered mood, and reduced wellbeing compared with those without sleep disorders. The results can help guide clinicians’ expectations of patient presentations in early psychosis clinical services.

## Method

### Participants

Sixty participants were recruited from 4 NHS Trusts: Oxford Health NHS Foundation Trust, Berkshire Healthcare NHS Foundation Trust, Central and North West London NHS Foundation Trust, and Northamptonshire Healthcare NHS Foundation Trust. The inclusion criteria were a diagnosis of nonaffective psychotic disorder; outpatient status; and aged between 18 and 30. Exclusion criteria were primary affective disorder; primary substance abuse disorder; organic or neurological disorder; and nonfluency in English. Diagnoses and current medication were taken from clinical notes. All participants provided written informed consent to take part in the study. The study received approval from an NHS Research Ethics Committee.

### Assessments

#### Diagnostic Interview for Sleep Disorders

The Diagnostic Interview for Sleep Patterns and Disorders (DISP^20^) was the primary sleep disorder assessment. This structured interview assesses a range of sleep disorders. Symptoms are rated according to diagnostic criteria, and severity was rated as mild, moderate, or severe based on the number and frequency of symptoms, duration of disorder, and the distress and impairment reported (see Supplementary Appendix 1 for the scoring algorithm). Diagnostic requirements were made as conservative as possible by including criteria from the DSM-5^19^, ICSD-2^21^, and ICSD-3^22^ classification systems, and requiring that all criteria must be satisfied for a positive diagnosis to be made. Dual ratings for diagnosis were carried out for 5 interviews, and inter-rater reliability calculated for a further 20 assessments. For some disorders (period limb movement syndrome, sleepwalking, REM sleep behavior disorder, narcolepsy, and hypersomnia) polysomnographic monitoring is required to confirm the diagnosis, therefore these are treated as positive screens rather than diagnoses. For sleep apnea, the number of symptom indicators endorsed is reported. For all other sleep disorders listed in table 1 self-reported symptoms, in the context of the interview, are sufficient to confirm diagnosis.

**Table 1.** Sleep Disorders Assessed by the Diagnostic Interview and Their Major Symptoms

Sleep Disorder		Description^a^
Circadian disorder	Advanced sleep-wake phase disorder	Advance of the sleep period in relation to desired sleep and wake time, resulting in inability to stay awake until desired time or wake up at desired time.
	Delayed sleep-wake phase disorder	Delay of the sleep period in relation to desired sleep and wake time, resulting in inability to stay awake until desired time or wake up at desired time.
Insomnia		Difficulty initiating or maintaining sleep, or sleep that is chronically non-restorative or poor in quality despite adequate opportunity for sleep.
Restless leg syndrome (RLS)		An urge to move the legs (often accompanied by unpleasant sensations) that gets worse in the evening, is relieved by movement, and disrupts sleep.
Periodic limb movement syndrome (PLMS)^b,c^		Repeated and highly stereotyped limb movements (eg, kicking during the night), associated with daytime fatigue.
Bruxism		Tooth grinding or clenching during sleep, can result in jaw pain or headaches on wakening.
Sleep apnea^d^		Characterized by pauses in breathing or shallow breathing through the night, commonly associated with snoring and resulting in daytime sleepiness and nonrestorative sleep.
Sleep walking^b^		Ambulation occurring during sleep—while sleep walking individuals may carry out inappropriate, nonsensical, or dangerous behaviors.
Night terrors		Sudden episodes of terror during sleep, distinguished from nightmares by amnesia for the episode and a lack of content recall (occurs in NREM sleep).
Enuresis		Involuntary voiding of bladder during sleep in individual older than 5 years of age.
REM sleep behavior disorder^b^		Lack of muscle atonia during REM sleep results in dream activity being carried out, often causing injury or other disruption.
Nightmare disorder		Recurrent episodes of waking from sleep with recall of intensely disturbing dream content involving dysphoric emotions (fear, anxiety, anger, sadness).
Sleep paralysis^c^		Recurrent inability to move limbs and trunk at onset or on waking from sleep, often accompanied by disturbing imagery or sensations.
Sleep-related hallucinations^c^		Recurrent hallucinations just before sleep onset or upon awakening, often visual.
Excessive sleepiness disorders	Narcolepsy with cataplexy^b^	Excessive sleepiness and sleep attacks on most days, accompanied by episodes of cataplexy (sudden and transient episodes of loss of muscle tone).
	Narcolepsy without cataplexy^b^	Excessive sleepiness and sleep attacks on most days, without cataplexy.
	Hypersomnia^b^	Excessive sleepiness on most days, accompanied by a lengthened sleep (≥11 h asleep in 24-h period).

*Note*: REM, rapid eye movement sleep; NREM, non-rapid eye movement sleep.

^a^Based on International Classification of Sleep Disorders—Third edition.

^b^Positive screen rather than diagnosis, other diagnostic tests (eg, polysomnography) required by diagnostic criteria.

^c^Assessment and treatment data not collected within interview.

^d^Symptoms reported rather than positive screen/diagnosis, further data required for positive screen and polysomnography for diagnosis.

If participants met criteria for diagnosis or positive screen for a particular disorder, they were then asked (*a*) if they have discussed the sleep problem with a medical professional and (*b*) if they are receiving treatment. Any treatment was then scored according to whether it was a recommended treatment or a nonrecommended treatment according to NICE Guidance or other published clinical recommendations—eg, for insomnia^23^ or nightmare disorder.^24^

#### Sleep Recording

##### Sleep Diary

**Participants completed a Consensus Sleep Diary^25^ for 7 days. Average sleep duration, sleep onset and offset, and sleep efficiency (the percentage of time in bed spent asleep) were calculated from the sleep diaries. Where less than 3 days of the diary were filled then the data were excluded from analysis.

##### Actigraphy

**Participants wore a wrist-based activity monitoring device (CamNTech Motionwatch 8) for the same 7 days to allow objective recording of activity, which provides estimates of sleep variables. Actigraphic data were analyzed within the proprietary software package (Motionware V1.1.25, CamNTech). Where less than 3 days of actigraphy data were available then the data were excluded from analysis. Actigraphic data were also checked against the diary, and where actigraphic data deviated significantly from the diary the actigraphic data were excluded from all further analyses. Each sleep period was analyzed by entering the time to bed and time out of bed from the sleep diary. If there were no diary data for a particular day visual inspection was applied to find the most likely sleep window—this process was used for a maximum of 3 nights per participant. In both cases, the software algorithm uses movement data to estimate the time spent asleep within the window, from which it calculates the sleep onset and offset, sleep duration, and sleep efficiency.

#### Sleep-50

A subgroup (*n* = 29) completed the Sleep-50.^26^ These data were used to validate DISP outcomes (Supplementary Appendix 2). The Sleep-50 is a self-report measure comprised of a number of subscales assessing particular sleep disorders as according to DSM-IV (TR).^27^ Agreement was assessed by testing if the total score in the relevant Sleep-50 subscale was higher in those receiving the DISP diagnosis vs those without the DISP diagnosis for that particular sleep disorder. This was testable for insomnia, nightmare disorder, restless leg syndrome (RLS), circadian disorder, and sleep walking. For insomnia and nightmare disorder, the relevant Sleep-50 subscale score was significantly higher for those receiving a diagnosis from the DISP (*P* = .003 and *P* = .001). The Sleep-50 subscale score was not significantly higher for RLS, circadian disorder or sleep walking in those receiving the respective DISP diagnoses. For RLS, this does appear to be a genuine lack of concordance between these measures (possibly due to the Sleep-50 indexing PLMS in the same subscale as RLS), but in the latter 2 cases the diagnostic groups were likely too small to detect differences (both *n* = 2).

#### Psychiatric Symptoms

Psychotic experiences were assessed with the Specific Psychotic Experiences Questionnaire^28^ (SPEQ), a self-report measure assessing the past month. The subscales for paranoia, hallucinations, cognitive disorganization, and grandiosity were used in the current study. The paranoia subscale is formed of 15 statements rated by the participant for frequency of the thought from 0 (“not at all”) up to 5 (“nearly all the time”), with a maximum possible score of 75. The subscale for hallucinations is comprised of 9 items rated on the same scale to comprise a maximum score of 45. For cognitive disorganization, 11 statements are marked as “yes” (this does apply to me) or “no” (this doesn’t apply to me), scored as 1 and 0, respectively. The grandiosity subscale is formed of 8 statements rated for agreement by the participant on a 0 (“not at all”) to 3 (“completely”) scale, with a maximum score of 24. Higher scores indicate more severe psychotic experiences.

Depression and anxiety were assessed using 2 subscales from the Depression Anxiety and Stress Scale (DASS-21),^29^ a self-report measure assessing the past month. The 7 items for each subscale are rated from 0 (did not apply to at all) to 3 (applied to me very much). Higher scores indicate higher levels of anxiety and depression.

#### Psychological Wellbeing

Health-related quality of life was assessed with the EQ-5D-5L.^30^ This self-report questionnaire assesses 5 health-related domains (mobility, self-care, ability to do usual activities, pain or discomfort, and anxiety and depression). Participants rate on a 1–5 scale their extent of issues in each domain (where 1 is no problems, 5 is extreme problems). This score is then entered into an algorithm which weights the responses by domain and population norms to give a decimal score (between 0 and 1), where higher values indicate higher health-related quality of life.

Fatigue was measured via the Multi-Dimensional Fatigue Symptom Inventory (MFSI) short form.^31^ This scale comprises 30 items in 5 subscales (general, emotional, physical, mental, vigor). Each item is rated from 0 (“not at all”) to 4 (“extremely”) by the participant. The total score for the questionnaire is the sum of the general, emotional, physical, and mental subscale scores minus the vigor subscale score. The maximum score is 100, with a greater score indicating greater fatigue.

### Analysis

All data were analyzed with SPSS 23.^32^ Descriptive variables from the diagnostic interview were reported. These are supplemented by statistics from the diary and actigraphic recordings (sleep duration, onset/offset, and efficiency) where recording is required in the diagnostic criteria, ie, circadian disorders and hypersomnia. Differences in sleep recording data were also analyzed for those with and without insomnia to support the interview-reported symptoms. Differences in psychotic experiences, mood, and wellbeing between those with and without sleep disorders were assessed via independent samples *t*-tests. Secondary analyses tested medication differences between those with and without sleep disorders. All hypothesis testing was two-tailed.

## Results

### Demographics

The participant group comprised a higher proportion of men (*n* = 39, 65%) than women (*n* = 21, 35%). The average age was 23.7 years old (SD = 3.2). Twenty-six participants (43.3%) were unemployed. Most participants were referred from early intervention psychosis services (*n* = 45, 75.0%). The most common diagnoses were psychosis (not otherwise specified) (*n* = 25, 41.7%) and schizophrenia (*n* = 17, 28.3%). The majority were prescribed antipsychotic medication (*n* = 49, 81.7%), and 40% (*n* = 24) were prescribed antidepressants. A detailed breakdown of demographics is available in table 2.

**Table 2.** Demographic Statistics for Study Group

Age—mean (SD)	23.7 (3.2)
Gender—*n* (%)	
Male	39 (65.0)
Female	21 (35.0)
Ethnicity—*n* (%)	
White/White British	30 (50.0)
Mixed/multiple ethnic groups	7 (11.7)
Black/African/Caribbean/Black British	11 (18.3)
Asian/Asian British	10 (16.7)
Other	2 (3.3)
Occupational status—*n* (%)	
Unemployed	26 (43.3)
Part time (inc on sick leave)	9 (15.0)
Full time (inc on sick leave)	7 (11.7)
Student	8 (13.3)
Volunteer	7 (13.3)
Homemaker	2 (3.3)
Diagnosis—*n* (%)	
Schizophrenia	17 (28.3)
Schizoaffective disorder	4 (6.7)
Schizophreniform psychosis	1 (1.7)
Psychosis not otherwise specified	25 (41.7)
First-episode psychosis	12 (20.0)
Team—*n* (%)	
Adult mental health team	15 (25.0)
Early intervention in psychosis service	45 (75.0)
Trust—*n* (%)	
OHFT	39 (65.0)
CNWL	11 (18.3)
BHFT	8 (13.3)
NHFT	2 (3.3)
Medication	
Antipsychotics—DDD (SD)	0.88 (0.8)
Not prescribed antipsychotics—*n* (%)	11 (18.3)
Antidepressants—DDD (SD)	0.71 (1.1)
Not prescribed antidepressants—*n* (%)	36 (60)
Dependent variables—mean (SD)	
Paranoia (SPEQ)	29.9 (22.2)
Hallucinations (SPEQ)	14.6 (11.8)
Cognitive Disorganization (SPEQ)	6.5 (3.2)
Grandiosity (SPEQ)	7.7 (6.2)
Depression (DASS)	8.6 (5.3)
Anxiety (DASS)	9.5 (6.3)
Fatigue (MFSI)	32.2 (24.1)
Health related quality of life (EQ-5D-5L)	4.0 (3.0)

*Note*: OHFT, Oxford Health NHS Foundation Trust; CNWL, Central and North West London NHS Foundation Trust; BHFT, Berkshire Healthcare NHS Foundation Trust; NHFT, Northampton Healthcare NHS Foundation Trust; DDD, defined daily dose; SPEQ, Specific Psychotic Experiences Questionnaire; DASS, Depression Anxiety Stress Scale; EQ-5D-5L, EuroQol 5 dimension 5 level scale; MFSI, Multdimensional Fatigue Symptom Inventory (Short Form).

### Sleep Disorders: Prevalence and Severity

Sleep disorders were common, with 80% (*n* = 48) of the participants receiving a positive screen or diagnosis for at least one disorder. As can be seen in table 3, the most common sleep diagnoses were insomnia (*n* = 30, 50%) and nightmare disorder (*n* = 29, 48.3%). However, there was a broad range of sleep issues presenting in this group and comorbidity was high, with an average of 3.3 sleep disorders per patient. Notable areas of comorbidity include insomnia and nightmares, with 20 individuals (33%) receiving a diagnosis of both disorders. The majority of all sleep disorders (*n* = 77, 52%) were severe in their presentation.

**Table 3.** Prevalence, Severity, and Treatment of Sleep Disorders Within the Study Group

Disorder	Diagnosis, %	Diagnosis, *n*	Kappa (Interrater)	Severity^b^—*n* (%)			Treatment^c^—*n* (%)		
				Mild	Moderate	Severe	None	Recommended	Other
Insomnia	50.0	30	0.765	2 (6.7)	11 (36.7)	17 (60.0)	14 (46.7)	6 (20)	10 (33.3)
Nightmare disorder	48.3	29	1	2 (6.9)	11 (37.9)	16 (55.2)	20 (71.4)	2 (7.1)	6 (21.4)
Sleep-related hallucinations	41.7	25	0.912	2 (8.0)	7 (28.0)	16 (64.0)	n/a	n/a	n/a
Excessive sleepiness disorders	23.3	14	0.773	0 (0.0)	5 (35.7)	9 (64.3)	11 (78.6)	—	3 (21.4)
RLS	23.3	14	0.886	4 (28.6)	3 (21.4)	7 (50.0)	13 (92.9)	—	1 (7.14)
PLMS	20.0	12	0.771	n/a	n/a	n/a	n/a	n/a	n/a
Bruxism	18.3	11	1	5 (45.5)	3 (27.3)	3 (27.3)	7 (63.6)	4 (36.4)	—
Sleep paralysis	15.0	9	1	1 (11.1)	5 (55.6)	3 (33.3)	n/a	n/a	n/a
Night terror	8.3	5	0.832	0 (0.0)	1 (20.0)	4 (80.0)	4 (80)	—	1 (20.0)
Circadian	8.3	5	0.950^a^	1 (20.0)	3 (60.0)	1 (20.0)	5 (100)	—	—
Sleep walking	5.0	3	1	n/a	n/a	n/a	3 (100)	—	—
REMSBD	3.3	2	0.900^a^	2 (100.0)	0 (0.0)	0 (0.0)	2 (100)	—	—
Enuresis	1.7	1	1	0 (0.0)	0 (0.0)	1 (100.0)	—	1 (100.0)	—
Any sleep disorder	80	48		—	—	—			
Total	—	160		20 (13.5)	51 (34.5)	77 (52.0)	79 (69.9)	13 (11.5)	21 (18.6)

*Note:* RLS, restless leg syndrome; PLMS, periodic limb movement syndrome; REMSBD, REM sleep behavior disorder; n/a, not applicable.

^a^Proportion agreement rather than kappa: used where responses from one rater were constant.

^b^Total *n* = 148; n/a for PLMS and sleep walking as severity can’t be rated from interview.

^c^Total *n* = 113; n/a for disorders where treatment not assessed within interview (sleep hallucinations, sleep paralysis, PLMS).

A large proportion of participants (*n* = 54, 90%) endorsed at least one apnea symptom (Supplementary Appendix 3). “Feeling sleepier than others your age” was the most commonly endorsed indicator (*n* = 42, 70%), although breathing related apnea symptoms were also present, with 17 participants (28.3%) reported waking from sleep not breathing or gasping or choking, and 9 participants (15%) reported stopping breathing or breathing abnormally while asleep.

#### Sleep Disorders and Sleep Recording Variables

For circadian disorders, sleep recordings were used to confirm shifted sleep times. Two individuals fulfilled the early sleep onset criterion (9 pm or earlier), with average sleep onsets of 19:34 and 20:30. Delayed sleep phase disorder required evidence of a late onset (1am or later). Three individuals satisfied this criterion with sleep onset averages of 01:36, 02:05, and 03:08. An 11 hour or more sleep duration (in a 24-hour period) was required to satisfy the hypersomnia diagnostic requirement, which was confirmed for 5 participants, with durations ranging from 11 hours 00 minutes to 12 hours 13 minutes. All times reported are from sleep diaries, but were corroborated by actigraphic recording.

Sleep recording differences are not required for a diagnosis of insomnia, but self-reported sleep duration was significantly lower in individuals with insomnia compared with those without insomnia, as measured by sleep diaries (8 h 12 min compared with 9 h 56 min, *t* = 5.09, *P* < .001) and actigraphy (7 h 25 min compared with 8 h 20 min; *t* = 2.57, *P* = .013). Individuals with insomnia disorder reported a lower sleep efficiency in their sleep diaries than those without insomnia (74.8% of time in bed was spent asleep in those with insomnia, compared with 89.4% in those without insomnia; *t* = 4.36, *P* < .001). Differences in actigraphic sleep efficiency were nonsignificant (*t* = 0.594, *P* = .556) in those with insomnia (75.3%) compared with those without insomnia (76.5%).

### Clinical Service Assessment and Treatment

Approximately half of all sleep disorders had been discussed with a medical professional (*n* = 60, 53.1%), with insomnia the most frequently discussed (80%, *n* = 24). The remaining disorders had either not been discussed (*n* = 43, 38.1%) or the participant was unsure if they had discussed it with a medical professional (*n* = 10, 8.8%). Night terror, nightmare disorder, and RLS were the disorders least commonly discussed with a clinician (all below 50%). Further information can be found in Supplementary Appendix 4.

Thirty-four sleep disorders had received treatment; this is 30% of all sleep disorders, or 56.7% of those that were discussed with a medical professional. The majority received a nonrecommended treatment (*n* = 21, 61.8%), typically antipsychotic or antidepressant medication prescribed with intent to aid in sleep. Of the 13 disorders which received recommended treatment, only hypnotic medication for insomnia (5 cases) was accessed through routine mental health care. Four instances of Bruxism were treated by a dentist, an enuresis case by surgery, and 2 nightmare disorder cases and 1 insomnia case received CBT interventions via randomized controlled trials conducted by our specialist research team.

### Psychotic Experiences, Affect, and Wellbeing

Compared with not having a sleep disorder diagnosis, having at least one sleep disorder was significantly associated with more severe paranoia, hallucinations, and cognitive disorganization (table 4). There was no significant relationship between sleep disorder presence and grandiosity.

**Table 4.** Differences in Psychotic Experiences, Mood, and Wellbeing in Those With a Sleep Disorder Compared With Those Without a Sleep Disorder

Dependent variable	Group Means (SD)		Independent Samples *t*-Test	
	No Sleep Disorder (*n* = 12)	Sleep Disorder (*n* = 48)	*t*-Value	*p*-Value
Psychotic experiences				
Paranoia (SPEQ)	16.83 (14.3)	33.15 (22.8)	−3.094	.005**
Hallucinations (SPEQ)	5.75 (4.9)	16.75 (12.1)	−4.898	.009**
Cognitive disorganization (SPEQ)	3.08 (2.6)	7.30 (2.7)	−4.784	.008**
Grandiosity (SPEQ)	5.84 (4.1)	8.19 (6.5)	−1.554	.132
Mood				
Depression (DASS)	4.08 (3.9)	9.59 (5.0)	−3.616	<.001***
Anxiety (DASS)	3.83 (3.43)	10.96 (6.0)	−5.407	.017*
Wellbeing				
Quality of life (EQ-5D-5L)	0.58 (0.2)	0.82 (0.2)	3.373	.001**
Fatigue (MFSI)	15.10 (16.7)	36.17 (23.9)	−3.437	.002**

*Note:* SPEQ, Specific Psychotic Experiences Questionnaire; DASS, Depression Anxiety Stress Scale; EQ-5D-5L, EuroQol 5-dimension 5 level scale; MFSI, Multidimensional Fatigue Symptom Inventory (Short Form).

*P < .05, **P < .01, *** *P* < .001.

Individuals with at least one sleep disorder had more severe depression and anxiety. They also reported significantly lower health-related quality of life, and higher fatigue.

### Medication and Sleep Disorders

Considering the number of individuals identifying their antipsychotic medication as a treatment for their sleep disorder we performed secondary analyses to test if antipsychotic medication dosage differed between those with and those without the most common sleep disorders (insomnia and nightmares). The defined daily dose (DDD) of antipsychotics in individuals with insomnia (average DDD = 0.72, SD = 0.59) was lower than in individuals without an insomnia diagnosis (1.05, 0.94), however this difference was nonsignificant (*t* = 1.617, *P* = .111). There was similarly no significant difference in antipsychotic DDD in individuals with nightmare disorder (average DDD = 0.81, SD = 0.62) compared with those without nightmare disorder (0.95, 0.94; *t* = 0.675, *P* = .503).

## Discussion

We assessed sleep disorders in 60 patients with early nonaffective psychosis. Strikingly, four-fifths of the patients were found to have a comorbid sleep disorder. These were not minor sleep issues—the majority of disorders were rated as severe in their chronicity, frequency, and distress or impairment. In over two-thirds of cases, no treatment for the sleep disorder was reported; in most of these instances the sleep problem had not been discussed with the care team. Even when treatment had been received, it was rarely the recommended treatment for the sleep disorder. The patients with sleep disorders had more severe paranoia, hallucinations, cognitive disorganization, depression, and anxiety. Quality of life was lower in patients with comorbid psychosis and sleep disorder. If the results generalize to the wider population of patients with psychosis, then clinical services need to give a greater priority to sleep than they have to date.

Diagnostic systems such as DSM-5 recommend that sleep problems should be assessed and treated irrespective of other psychiatric comorbidities.^19^ This does not appear to be happening in psychosis services. When sleep is assessed in individuals with psychosis, many clinicians report only doing so informally^5^—this may mean that the depth, complexity, and impact of sleep issues are not revealed. The use of brief screening measures such as the Insomnia Severity Index^33^ or Disturbing Dreams and Nightmares Severity Index^34^ may be of use in facilitating these assessments in clinical practice. Expanding education on sleep disorders within training pathways may also help to address the neglect of these issues.

Provision of treatment for sleep problems is another issue raised by these findings. Where treatment had been given it was often a nonrecommended treatment, eg, antipsychotic medication or sleep hygiene advice for insomnia, neither of which have been shown to be effective.^35,36^ The primary recommended treatment for insomnia (CBT) has been shown to be effective in this patient group,^4^ and adaptations to the treatment for individuals with psychosis are available,^37^ but none of our study participants received this treatment from mental health services. Hypnotic medication was the only recommended sleep intervention received from mental health services. Insomnia treatment guidelines do include hypnotic medication, but they state that it should be reserved for acute phases of insomnia only,^23^ due to side effects (including cognitive impairment and increased risk of vehicular accidents) and limited effectiveness once tolerance develops.^38^

Patient factors have previously been cited as a barrier to clinicians referring patients for treatment.^5^ However, it has been shown that patients are very much able to engage with treatment for insomnia. This includes outpatients with current delusions and hallucinations^12^ and inpatients who were acutely unwell.^39^ Many patients highly value the sleep treatment they receive,^4^ and our clinical experience is that treating sleep problems results in higher engagement with other clinical interventions. The other issue cited by clinicians is availability of treatment,^5^ which does provide a greater barrier to access even when the treatments have been highly manualized.

Insomnia was the most prevalent sleep disorder but nightmare disorder was also found to be common, often severe, yet predominantly not discussed with the care team. A recent student survey found that only 11% of individuals with severe nightmares had discussed them with a health care provider, and those who believed nightmare disorders were untreatable (67.3%) were less likely to report them.^40^ It is possible that a lack of awareness regarding the treatability of nightmares might also affect reporting by individuals with psychosis. Although effective treatments for nightmare disorders do exist,^24,41^ they have not yet been tested in this clinical group, although such a trial is underway.^42^

### Limitations

The first key issue is the representativeness of the participant group. It is simply unknown how representative the participants are of the wider population of patients with nonaffective psychosis. The study group could, eg, be biased as individuals with sleep problems may have been more motivated to take part. It is worth noting that the insomnia prevalence in this study (50%) is higher than has been reported a larger study (29%, sample *n* = 623^43^). The current study was advertised as recruiting “good and bad sleepers” to attempt to minimize this bias, and indeed there were a number of individuals with no sleep issues. The demographic data also indicate that our participant group is reasonably representative of those seen within early intervention services in age, gender, and local ethnicity.^44^ Given the importance of this issue to patients, and the relative ease of sleep assessment, larger studies should attempt to assess sleep disorders to test if the results are representative of the sleep disorders in the population.

Our screening of several sleep issues is limited due to a lack of polysomnography (PSG). Sleep apnea is a key disorder which we were not able to screen for using the DISP items, and were not able to diagnose due to lack of PSG. Future studies should either use PSG or apnea specific screening measures (eg, STOP-BANG^45^) to further investigate the high prevalence of apnea symptoms reported in this study and others.^46^ Polysomnography is also required to provide a confirmed diagnosis for several sleep disorders in this study (eg, PLMS, sleep walking, hypersomnia). Even so, the structured interview and sleep recording used in the current study allowed diagnosis or positive screens to be provided for the majority of sleep disorders, and is more likely than PSG to be available in typical mental health services.

However, it is worth noting that PSG might provide a differential diagnosis in cases where a symptom (eg, excessive sleepiness) might result from multiple disorders. Further work should investigate differential diagnosis of sleep issues in this participant group, where there is clear comorbidity, to provide further clinical guidance. Another issue is that for several sleep disorder diagnoses (eg, hypersomnia, night terrors, sleep walking) the symptoms need to “not be better explained by another sleep disorder, medical or neurological disorder, mental disorder, medication use, or substance use disorder.”^22^ This raises the question of which (if any) of these diagnoses might be excluded by having a psychotic disorder diagnosis or being in receipt of antipsychotic medication.

The frequency of sleep-related hallucinations is high in our study. In part, this reflects a phenomenological overlap between hallucinations in the context of psychosis and sleep-related hypnagogic or hypnopompic hallucinations. Some participants described the hypnagogic hallucinations as being different in character (eg, more visual) than their daytime hallucinations, or only had sleep-related hallucinations (with no daytime hallucinations)—but in many cases there was no clear divide. Issues in discriminating between these phenomena have been noted in the narcolepsy literature,^47^ but further research is clearly needed to investigate the overlap in individuals with psychosis.

Medication use is potentially an important factor in considering sleep in individuals with psychosis. Antipsychotic medication may improve sleep—however daytime sedation is common, and antipsychotics may themselves increase risk of certain sleep issues (eg, sleep apnea^29^). The picture is complicated by antidepressants, which have their own interaction with sleep^48^ and are often prescribed alongside or instead of antipsychotics. Our secondary medication analyses indicate that antipsychotic medication dosage did not differ between those with or without an insomnia or nightmare disorder diagnosis, indicating medication is not a significant factor in sleep disorders in our study group, but further research is needed.

Lastly, this study is cross-sectional, therefore we are not able to investigate the direction of effect between sleep disorders and psychotic experiences, mood, or wellbeing. Longitudinal studies to investigate the temporal relationship between sleep disorders and psychotic experiences in clinical populations would therefore be valuable. Nevertheless, there is increasing evidence pointing to sleep disruption as a contributory causal factor in psychosis.^1,17,18^ This means that there is a possibility that by improving sleep it may be possible to improve psychosis, representing an exciting new treatment target.^18^ However, independent of any relationship with psychotic experiences, our view is that the assessment and treatment of sleep disorders among those with psychosis merits greater clinical attention.

## Funding

S.R. is supported by a Medical Research Council Doctoral Studentship and a Balliol College Dervorguilla Scholarship (University of Oxford). B.S. is supported by a Wellcome Trust Strategic Award (098461/Z/12/Z) to the Sleep and Circadian Neuroscience Institute (SCNi). D.F. is a grant holder of the Wellcome Trust Strategic Award and is supported by a National Institutes of Health Research (NIHR) Professorship (grant number RP-2014-05-003). This research study was supported by the NIHR Oxford Health Biomedical Research Centre. The views expressed are those of the authors and not necessarily those of the NHS, the NIHR, or the Department of Health.

## Supplementary Material

Appendix 1

Appendix 2
