# Supplementary material for: Sleep Disorders in Early Psychosis: Incidence, Severity, and Association With Clinical Symptoms
Source: Schizophr Bull. 2018 Sep 8;45(2):287–95. doi: 10.1093/schbul/sby129 (PMC6403049; doi:10.1093/schbul/sby129)
Supplement: Appendix 1 [file sby129_suppl_appendix-1.doc]

| **Variable** | **Coding** | **Reflections on notes/coding** |
| --- | --- | --- |
| Diagnosis | 0 = No Diagnosis  1 = Diagnosis  -99 = Unknown/Missing  All diagnostic criteria to be satisfied for a 1  **Exceptions** (where not possible to tell diagnosis without polysomnography or other tests). PSG criteria shaded in grey in table below  PLMS: only have presence/absence of kicking  REM sleep behaviour disorder: only have reports of activity during night  Narcolepsy (with or without cataplexy): requires multiple sleep onset latency test (MSLT) with polysomnography (PSG) to confirm sleepiness, or hypocretin tests for narcolepsy with cataplexy  Hypersomnia: requires multiple sleep onset latency test with polysomnography to confirm sleepiness  Apnea: symptom items are not sufficient for positive screen (no BMI or neck circumference available for STOP-BANG), PSG required for diagnosis. Symptom index reported instead.  NB Bruxism: some supplementary criteria require PSG, but diagnosis possible without | DISP originally has 0-3 coding (0 =not present, 1= symptoms only, 2= subclinical, 3=diagnosis), but this was changed to 0/1 to simplify reporting  Diagnostic criteria are derived from ICSD-2, DSM-5, and ICSD-3 in order to encompass maximum information  Only current sleep disorders were included. |
| Severity | Severity coded on 1-3 scale (1=mild, 2=moderate, 3=severe) composed individually for each disorder based on below characteristics:  1) symptom counts (where extra indicators beyond diagnosis are available)  2) frequency of symptoms (where threshold from diagnostic criteria is available)  3) duration of symptoms (where threshold from diagnostic criteria is available)  4) distress/impairment rated on 1-10 scale, highest used – rated to severity as following: mild = 1-3; moderate = 4-6; severe =>7  Overall coding is based on average of these four characteristics – where indicators are split across two levels, round down e.g. D/I and frequency is 3, duration and symptom counts are 2, score would be 2 | DISP originally requires 1-3 scoring based on hospitalisations and occupational/functional issues, but this requirement is unrealistic for psychosis where individuals are often unemployed, and where hospitalisations that have occurred are difficult to place on sleep issues. |
| Treatment | 0 = no  1 = Recommended treatment from care team (e.g. for insomnia: Cognitive Behavioural Therapy for Insomnia or hypnotic medication)  2 = Non-recommended treatment from care team (e.g. antipsychotics, sleep hygiene sheet, non-specific sleep advice)  3 = self treated  -99 = unknown | For each disorder where screening questions passed, participant is asked if they have mentioned the problem to their care team, and then if they are receiving treatment for it |

## Appendix I: Scoring guidelines for the diagnostic interview for sleep patterns and disorders (DISP)

**Other notes**

If > or <, anchor number was used (e.g. >10, used 10); If range given average was used

| **Disorder** | **Diagnostic criteria – from ICSD2 unless labelled otherwise** | **Scoring - Diagnosis** | **Scoring - Severity** |
| --- | --- | --- | --- |
| **Circadian = Delayed or Advanced sleep phase syndrome** | | | |
| Delayed sleep phase syndrome | A. There is a delay in the phase of the major sleep period in relation to the desired sleep time and wake-up time, as evidenced by a chronic or recurrent complaint of inability to fall asleep at a desired conventional clock time together with the inability to awaken at a desired and socially acceptable time.  B. When allowed to choose their preferred schedule, patients will exhibit normal sleep quality and duration for age and maintain a delayed, but stable, phase of entrainment to the 24-hour sleep-wake pattern.  C. Sleep log or actigraphy monitoring (including sleep diary) for at least seven days demonstrates a stable delay in the timing of the habitual sleep period.  D (from ICSD3) difficulty causes sleepiness, insomnia, or both | A: Y to “strong night owl”, regularly asleep later than 1am AND difficulty falling asleep at earlier times  B: unable to assess due to high level of sleep disorder co-morbidity in the group  C: sleep diary indicates consistent late sleep time i.e. post 1am sleep, actigraph confirms  D: having great difficulty waking up in the morning (sometimes/always) OR feeling awake and refreshed after sleeping (never/sometimes) | 1) 2hr out (i.e. 1am); mild distress/impairment  2) 4hr out (i.e. 3am); moderate distress/impairment  3) 6hr out (i.e. 5am); severe distress/impairment |
| Advanced sleep phase syndrome | A. There is an advance in the phase of the major sleep period in relation to the desired sleep time and wake-up time, as evidenced by a chronic or recurrent complaint of inability to stay awake until the desired conventional clock time, together with an inability to remain asleep until the desired and socially acceptable time for awakening.  B. When patients are allowed to choose their preferred schedule, sleep quality and duration are normal for age with an advanced, but stable, phase of entrainment to the 24-hour sleep-wake pattern.  C. Sleep logs or actigraphy monitoring (including sleep diaries) for at least seven days demonstrates a stable advance in the timing of the habitual sleep period.  D (from ICSD3) difficulty causes sleepiness, insomnia, or both | A: Y to “strong early bird”, regularly asleep before 9pm AND difficulty staying awake at later times  B: unable to assess due to high level of sleep disorder co-morbidity in the group  C: sleep diary indicates consistent early sleep time i.e. pre 9pm, actigraph confirms  D: having difficulty waking too early and unable to get back to sleep (yes/no) OR feeling awake and refreshed after sleeping (never/sometimes) | 1) 2hr out (i.e. 9pm); mild distress/impairment  2) 4hr out (ie. 7pm); moderate distress/impairment  3) 6hr out (i.e. 5pm); severe distress/impairment |
| Insomnia | A. Difficulty initiating sleep, difficulty maintaining sleep, or waking up too early or sleep that is chronically nonrestorative or poor in quality.  B. The above sleep difficulty occurs despite adequate opportunity and circumstances for sleep.  C. At least one of the following forms of daytime impairment related to the night time sleep difficulty is reported by the patient:  1. Fatigue or malaise  2. Attention, concentration, or memory impairment  3. Social or vocational dysfunction or poor school performance  4. Mood disturbance or irritability  5. Daytime sleepiness  6. Motivation, energy, or initiative reduction  7. Proneness for errors or accidents at work or while driving  8. Tension, headaches, or gastrointestinal symptoms in response to sleep loss  9. Concerns or worries about sleep  D: (from DSM-5) Trouble sleeping has occurred at least 3 times a week for the last 3 months | A: Y to difficulty initiating sleep OR staying asleep OR falling back to sleep OR waking too early in morning (DASLP103-106)  B: From routine sleep section there is adequate time in bed, plus no other obvious reason for lack of sleep, e.g. shift work or children.  C: One of consequences (fatigue, attention impairment, social dysfunction etc. – DASLP113-123)) endorsed OR distress and dmpairment > 0  D: Occurs >=3/wk, lasted >=3 months | 1) 1 of A; 3/wk; =3 mo; mild distress/impairment  2) 2 of A; >=4/wk; <= 1 year; moderate distress/impairment  3) 3-4 of A; >=6/wk; >1 year; severe distress/impairment |
| Obstructive Sleep Apnoea | OSA may be diagnosed if the AHI (apnoea-hypopnoea index) or REI (respirator index event) from PSG is ≥5 episodes/hour with evidence of respiratory effort during all respiratory events; diagnosis is not better explained by another sleep, medical, or neurological disorder, or medication or drug use; and at least 1 of the following is present:   1. Complaints of daytime sleepiness, unrefreshing sleep, fatigue, insomnia, or unintentional sleep episodes during wakefulness 2. Waking with breath holding, gasping, or choking 3. Bed partner reports loud snoring, breathing interruptions, or both, during patient's sleep | Not possible to identify from DISP screening items, additional data to check criteria from screening questionnaires (e.g. BMI or neck circumference for STOP-BANG) not available. | Number of the 6 symptom indicators (DASLP172-177) endorsed reported. |
| Restless leg syndrome | A. The patient reports an urge to move the legs, usually accompanied or caused by uncomfortable and unpleasant sensations in the legs.  B. The urge to move or the unpleasant sensations begin or worsen during periods of rest or inactivity such as lying or sitting.  C. The urge to move or the unpleasant sensations are partially or totally relieved by movement, such as walking or stretching, at least as long as the activity continues.  D. The urge to move or the unpleasant sensations are worse, or only occur, in the evening or night.  E. The condition is not better explained by another current sleep disorder, medical or neurological disorder, mental disorder, medication use, or substance use disorder.  F: (from ICSD-3) Must cause disruption to sleep, distress, or disturbance  G: (From DSM-5) Has to occur at least 3/week for at least last three months | A: Y to repeated urge to move legs (strange or uncomfortable feelings optional) (DASLP256)  B: Y to sensations occur mostly at rest (DASLP259)  C: Y to relieved by standing or walking around (DASLP260)  D: Y to worse late in day or in evening (DASLP261)  F: Disturbs sleep (DASLP262) OR distress OR impairment > 0  G:Onset >=3mo ago, Frequency >=3 wk | 1) =3/week; <=3 months; cause some difficulty getting to sleep (DASLP262); mild distress/impairment  2) <=5/week; <=1yr; cause great difficulty getting to sleep(DASLP262); moderate distress/impairment  3) >6week; >1yr; caused great difficulty getting to sleep(DASLP262); severe distress/impairment |
| Periodic limb movement | A. Polysomnography demonstrates repetitive, highly stereotyped, limb movements that are:  1. 0.5 to five seconds in duration  2. Of amplitude greater than or equal to 25% of toe dorsiflexion during calibration  3. In a sequence of four or more movements  4. Separated by an interval of more than five seconds (from limb-movement onset to limb-movement onset) and less than 90 seconds (typically there is an interval of 20 to 40 seconds)  B. The PLMS Index exceeds five per hour in children and 15 per hour in most adult cases.  C. There is clinical sleep disturbance or a complaint of daytime fatigue.  D. The PLMS are not better explained by another current sleep disorder, medical or neurological disorder, mental disorder, medication use, or substance use disorder. | Y to kicking at night (DASLP313) | All criteria require PSG therefore not scorable beyond report of kicking |
| Bruxism | A. The patient reports or is aware of tooth-grinding or tooth clenching during sleep.  B. One or more of the following is present:  1. Abnormal wear of the teeth.  2. Jaw muscle discomfort, fatigue, or pain and jaw lock upon awakening  3. Masseter muscle hypertrophy upon voluntary forceful clenching.  4. Sounds associated with bruxism.  C. The jaw muscle activity is not better explained by another current sleep disorder, medical or neurological disorder, medication use, or substance use disorder. | A: Y to grinding/clenching teeth at night (DASLP228)  B: Y to dentist noticing teeth worn down OR waking in morning with headache and sore jaw (DASLP229-230) | 1) 2 of DASLP228-230; no dental problems (DASLP236); mild distress or impairment  2) 3 of DASLP228-230; dental problems (DASLP236236); moderate distress or impairment  2) 3 of DASLP228-230; dental problems (DASLP236236); severe distress or impairment |
| Sleep walking | A. Ambulation occurs during sleep.  B. Persistence of sleep, an altered state of consciousness, or impaired judgement during ambulation is demonstrated by:  1. Difficulty in arousing the person  2. Mental confusion when awakened from an episode  3. Amnesia (complete or partial) for the episode  4. Routine behaviours that occur at inappropriate times  5. Inappropriate or nonsensical behaviours  6. Dangerous or potentially dangerous behaviours  C. The disturbance is not better explained by another sleep disorder, medical or neurological disorder, mental disorder, medication use, or substance use disorder. | A: Y to reporting sleep walking (DASLP437)  B: Y to all NREM indicators (e.g. no memory, difficult to wake, inappropriate behaviours -DASLP438-543) | Unable to rate:  No frequency/duration criteria  Distress/impairment not included as part of questionnaire |
| Night terrors | A. A sudden episode of terror occurs during sleep, usually initiated by a cry or loud scream that is accompanied by autonomic nervous system and behavioural manifestations of intense fear.  9  B. At least one of the following associated features is present:  1. Difficulty in arousing the person  2. Mental confusion when awakened from an episode  3. Amnesia (complete or partial) for the episode  4. Dangerous or potentially dangerous behaviours  C. The disturbance is not better explained by another sleep disorder, medical or neurological disorder, mental disorder, medication use, or substance use disorder. | A: Y to episodes of strong feelings of fear while sleeping (DASLP549) AND increase in heart rate/breathing etc. during episode (DASLP550);  B Y to all NREM indicators (DASLP551-553) | 1) <1wk; <1mo; mild distress/impairment  2) <7wk; <6mo; moderate distress/impairment  3) 7/wk; >=6mo; severe distress/impairment  *Severity and frequency based on nightmares |
| Enuresis | A. The patient is older than five years of age  B. The patient exhibits recurrent involuntary voiding during sleep, occurring at least twice a week.  C: the condition has been present for at least three months  D The patient has never been consistently dry during sleep (for primary)  OR the patient has previously been consistently dry during sleep for at least six months (for secondary) | A: all sample >18, all satisfied  B: Y to current problem with bedwetting (DASLP579 AND DASLP589), occurring >= 2 times a week  C: duration >= 3 months  D: not discriminating primary/secondary | 1) frequency 2/wk*; duration <6mo; mild distress/impairment  2) 3-4/wk; 6mo-1yr; moderate distress/impairment  3) >=5/wk; >=1yr; severe distress/impairment  *Frequency anchored on diagnostic criteria |
| REM sleep behaviour disorder | A. Presence of REM sleep without atonia: the EMG finding of excessive amounts of sustained or intermittent elevation of submental EMG tone or excessive phasic submental or (upper or lower) limb EMG twitching.  B. At least one of the following is present:  1. Sleep related injurious, potentially injurious, or disruptive behaviours by history  2. Abnormal REM sleep behaviours documented during polysomnographic  C. Absence of EEG epileptic form activity during REM sleep unless RBD can be clearly distinguished from any concurrent REM sleep related seizure disorder.  D. The sleep disturbance is not better explained by another sleep disorder, medical or neurological disorder, mental disorder, medication use, or substance use disorder. | Diagnosis:  A: Y to acting out dreams (DASLP450) AND move so much that could cause damage to self or partner (DASLP454) OR movements disrupt sleep (DASLP456) | 1) mild distress/impairment  2) moderate distress/impairment  3) severe distress/impairment  No frequency/duration criteria available in diagnostic manuals |
| Nightmares | A. Recurrent episodes of awakenings from sleep with recall of intensely disturbing dream mentation, usually involving fear or anxiety but also anger, sadness, disgust, and other dysphoric emotions.  B. Full alertness on awakening with little confusion or disorientation; recall of sleep mentation is immediate and clear.  C. At least one of the following associated features is present:  1. Delayed return to sleep after the episodes.  2. Occurrence of episodes in the latter half of the habitual sleep period  3. ICSD3 – causes clinical significant distress or impairment | A: Y to often having nightmares (DASLP612) AND feel frightened/angry/uneasy (DASLP613) OR make feel distressed (DASLP614)  B: Y to clearly recall (DASLP615)  C: Y difficulty falling back to sleep (DASLP616) OR >0 distress/impairment | 1) frequency <1wk; duration <=1mo; mild distress/impairment  2) >=1/wk; <6mo; moderate distress/impairment  3) 7/wk; =>6mo; severe distress/impairment  Severity based on DSM-5 guidelines |
| Sleep-related hallucinations | A: There is a complaint of recurrent hallucinations that are experienced just prior to sleep onset or upon awakening during the night or in the morning.  B. The hallucinations are predominantly visual.  C. The disturbance is not better explained by another sleep disorder (especially narcolepsy), mental disorder, medical disorder, medication, or substance use. | A Y to seeing or hearing things when falling asleep or waking up currently (DASLP399 AND DASLP409)  B: unknown | Indicators = unusual or bizarre; frightening; occurring on waking or after naps; see/hear when drowsy (DASLP400-403)  1) 1 indicator; frequency <=1/mo; mild distress/impairment  2) 2 indicators; <1/wk; moderate distress/impairment  3) 3-4 indicators; >=1wk; severe distress/impairment |
| Sleep paralysis | All ICSD3  A. A recurrent inability to move the trunk and all of the limbs at sleep onset or upon awakening from sleep.  B. Each episode lasts seconds to a few minutes.  C. The episodes cause clinically significant distress including bedtime anxiety or fear of sleep.  D. The disturbance is not better explained by another sleep disorder (especially narcolepsy), mental disorder, medical condition, medication, or substance use. | A Y to wake up unable to move as if paralysed (DASLP412) AND greater than 1 occurrence in last year (DASLP419)  B unknown  C >0 distress or impairment | 1) frequency <=1/mo; mild distress/impairment  2) < 1/wk; moderate distress/impairment  3) >=1/wk; severe distress/impairment  Frequency boundaries based on Sharpless et al 2010 |
| **Excessive sleepiness disorders = hypersomnia OR narcolepsy with OR without cataplexy** | | | |
| Narcolepsy with cataplexy | A. The patient has a complaint of excessive sleepiness occurring almost daily for at least three months.  B. A definite history of cataplexy, defined as sudden and transient episodes of loss of muscle tone  triggered by emotions, is present  C. The diagnosis of narcolepsy with cataplexy should be confirmed by nocturnal polysomnography followed by an MSLT. Alternatively, hypocretin-1 levels in the CSF are less than or equal to 110pg/ mL or one third of mean normal control values.  D. The hypersomnia is not better explained by another sleep disorder, medical or neurological disorder, mental disorder, medication use, or substance use disorder. | A: Y to excessive sleepiness >=4/wk (DASLP341) AND Y to sleep attacks >= 4/wk (DASLP348)  B: Y to cataplexy reports (DASLP365-394) | 1) mild distress/impairment  2) moderate distress/impairment  3) severe distress/impairment |
| Narcolepsy without cataplexy | A. The patient has a complaint of excessive sleepiness occurring almost daily for at least three months.  B. Typical cataplexy is not present, although doubtful or atypical cataplexy-like episodes may be reported.  C. The diagnosis of narcolepsy without cataplexy must be confirmed by nocturnal polysomnography followed by an MSLT.  D. The hypersomnia is not better explained by another sleep disorder, medical or neurological disorder, mental disorder, medication use, or substance use disorder | A: Y to excessive sleepiness >=4/wk (DASLP341) AND Y to sleep attacks >= 4/wk (DASLP348)  B: N to cataplexy reports (DASLP365-394) | 1) mild distress/impairment  2) moderate distress/impairment  3) severe distress/impairment |
| Hypersomnia | A: Extreme daytime sleepiness (>5/wk for >=3 mo)  B: no cataplexy  C: An MSLT performed according to standard techniques shows fewer than two sleep onset REM periods or no sleep onset REM periods if the REM latency on the preceding polysomnogram was less than or equal to 15 minutes.  D: one of  1. The MSLT shows a mean sleep latency of ≤ 8 minutes. OR  2. (ICSD3) >=11hr in 24hr sleep period as demonstrated by sleep log and confirmed by actigraphy | A = Y to excessive sleepiness >=4/wk (DASLP341)  B: N to cataplexy reports (DASLP365-394)  D = >=11hr sleep in 24 hours average reported in sleep diary, confirmed by actigraphy | indicators = always experiencing difficulty waking up (DASLP480c), never feeling awake and refreshed from sleeping (DASLP480d), feeling that sleeping too long (DASLP482)  1) duration 3-6mo; 1 of indicators; mild distress/impairment  2) >6mo; 2 of indicators; moderate distress/impairment  3) >=1yr; 3 indicators; severe distress/impairment |
