# Supplementary material for: Sleep Disorders in Early Psychosis: Incidence, Severity, and Association With Clinical Symptoms
Source: Schizophr Bull. 2018 Sep 8;45(2):287–95. doi: 10.1093/schbul/sby129 (PMC6403049; doi:10.1093/schbul/sby129)
Supplement: Appendix 2 [file sby129_suppl_appendix-2.doc]

**Appendix 2: Sleep-50 agreement with diagnostic interview**

| **Disorder** | **DISP diagnosis** | |  | **No DISP diagnosis** | | p-value^b^ |
| --- | --- | --- | --- | --- | --- | --- |
|  | N^a^ | Sleep-50 Subscale Average (SD) |  | N^a^ | Sleep-50 Subscale Average (SD) |  |
| Insomnia | 22 | 23.13 (5.7) |  | 7 | 15.0 (3.8) | 0.003 |
| Nightmare Disorder | 20 | 15.56 (2.6) |  | 9 | 6.33 (5.4) | 0.001 |
| Restless Legs Syndrome | 8 | 7.88 (2.4) |  | 21 | 6.60 (2.21) | 0.191 |
| Circadian Disorder | 2 | 8.0 (2.8) |  | 27 | 6.22 (2.4) | 0.326 |
| Sleep walking | 2 | 5.0 (1.4) |  | 27 | 3.59 (1.0) | 0.082 |

^a^Sleep-50 completed by n=29 subsample of n=60 participant group

^b^independent sample t-tests

**Appendix 3: Obstructive Sleep Apnea symptoms (OSA) in study group**

**Table A: Rates of individual apnea symptom endorsement**

| **Apnea items in DISP** | **Rate of endorsement - n (%)** |
| --- | --- |
| Do you feel that you are sleepier than others your age? | 42 (70.0) |
| Do you feel that your sleep is generally unrefreshing? | 35 (58.3) |
| Have you ever been told that you snore? | 24 (40.0) |
| Have you ever awoken from sleep not breathing, or gasping, or choking? | 17 (28.3) |
| Have you had times during sleep when you stop breathing or breathe abnormally? | 9 (15.0) |
| Have you ever been told that you stop breathing, gasp, or choke while you sleep? | 8 (13.3) |

**Table B: Sum of apnea items endorsed (symptom index)**

| **No. of apnea items endorsed** | **Frequency - n (%)** |
| --- | --- |
| 0 | 6 (10.0) |
| 1 | 16 (26.7) |
| 2 | 14 (23.3) |
| 3 | 13 (21.7) |
| 4 | 7 (11.7) |
| 5 | 4 (6.7) |
| 6 | 0 (0.0) |

**Appendix 4: Assessment of sleep disorders (by treatment)**

*Mention = “have you discussed [sleep problem] with a medical professional/your doctor or care team?”*

*Awareness by sleep disorder (t = treatment, m=mention, Y=yes, N=no)*

| **Diagnosis** | **YtYm** | **YtNm** | **NtYm** | **NtNm** | **Unsure** |
| --- | --- | --- | --- | --- | --- |
| **Insomnia** | 16 (53.3) | 3^a^ (10.0) | 8 (26.7) | 3 (10.0) | 0 (0) |
| **Nightmare Disorder** | 8 (28.6) | 0 (0) | 5 (17.9) | 16 (53.6) | 0 (0) |
| **RLS** | 1 (7.1) | 0 (0) | 4 (28.6) | 7 (50.0) | 2 (14.3) |
| **Brux** | 4 (36.4) | 0 (0) | 2 (18.2) | 4 (36.4) | 1 (9.1) |
| **Excessive sleep** | 3 (21.4) | 0 (0) | 4 (28.6) | 3 (21.4) | 4 (28.6) |
| **Night terror** | 1 (20.0) | 0 (0) | 0 (0) | 4 (80.0) | 0 (0) |
| **Circadian** | 0 (0) | 0 (0) | 3 (60.0) | 2 (50.0) | 0 (0) |
| **Sleep walking** | 0 (0) | 0 (0) | 0 (0) | 1 (33.3) | 2 (66.7) |
| **REMSBD** | 0 (0) | 0 (0) | 0 (0) | 1 (50.0) | 1 (50.0) |
| **Enuresis** | 1 (100) | 0 (0) | 0 (0) | 0 (0) | 0 (0) |
| **TOTAL** | 34 | 3 | 26 | 40 | 10 |

^a^self treated, entered as no treatment in paper; treatment is defined as being received from a medical professional
